# Modelling the impact of travel restrictions on COVID-19 cases in Hong Kong in early 2020

**DOI:** 10.1186/s12889-021-11889-0

**Published:** 2021-10-18

**Authors:** Wang-Chun Kwok, Chun-Ka Wong, Ting-Fung Ma, Ka-Wai Ho, Louis Wai-Tong Fan, King-Pui Florence Chan, Samuel Shung-Kay Chan, Terence Chi-Chun Tam, Pak-Leung Ho

**Affiliations:** 1 Department of Medicine, Queen Mary Hospital, Hong Kong, SAR China; 2 Department of Statistics, University of Wisconsin, Madison, USA; 3 Department of Astronomy, University of Wisconsin, Madison, USA; 4 Department of Mathematics, Indiana University, Bloomington, USA; 5 Department of Microbiology and Centre for Infection, University of Hong Kong, Hong Kong, SAR China

**Keywords:** Coronavirus disease 2019 (COVID-19), Border restriction, Susceptible exposed infectious recovered (SEIR) model

## Abstract

**Background:**

Coronavirus Disease 2019 (COVID-19) led to pandemic that affected almost all countries in the world. Many countries have implemented border restriction as a public health measure to limit local outbreak. However, there is inadequate scientific data to support such a practice, especially in the presence of an established local transmission of the disease.

**Objective:**

To apply a metapopulation Susceptible-Exposed-Infectious-Recovered (SEIR) model with inspected migration to investigate the effect of border restriction as a public health measure to limit outbreak of coronavirus disease 2019.

**Methods:**

We apply a modified metapopulation SEIR model with inspected migration with simulating population migration, and incorporating parameters such as efficiency of custom inspection in blocking infected travelers in the model. The population sizes were retrieved from government reports, while the number of COVID-19 patients were retrieved from Hong Kong Department of Health and China Centre for Disease Control (CDC) data. The *R*_0_ was obtained from previous clinical studies.

**Results:**

Complete border closure can help to reduce the cumulative COVID-19 case number and mortality in Hong Kong by 13.99% and 13.98% respectively. To prevent full occupancy of isolation facilities in Hong Kong; effective public health measures to reduce local *R*_0_ to below 1.6 was necessary, apart from having complete border closure.

**Conclusions:**

Early complete travel restriction is effective in reducing cumulative cases and mortality. However, additional anti-COVID-19 measures to reduce local *R*_0_ to below 1.6 are necessary to prevent COVID-19 cases from overwhelming hospital isolation facilities.

## Background

Since the outbreak of Coronavirus Disease 2019 (COVID-19) in late 2019, it rapidly evolved and became a pandemic. As of 30th August 2021, there were more than 216 million COVID-19 cases worldwide [1]. The reported global infection fatality rate was 0.15% in a systematic evaluations. To limit the scale of local disease outbreak, many countries implemented travel restrictions to countries experiencing COVID-19 countries despite the World Health Organization (WHO)‘s advice to the contrary [2]. Also, there is inadequate scientific data to support border restriction as a public health measure and it’s effectiveness in limiting local outbreak of an emerging infectious disease in the presence of an established local transmission. Whether border restriction can effectively limit local outbreak of COVID-19 is still debatable.

There has been great debate on the border restriction policy in Hong Kong since early 2020. On 23rd January 2020, Hong Kong confirmed its first imported case of COVID-19 from Hubei [3]. In the subsequent weeks, the number of imported cases rapidly rose despite initiation of various public health measures. Medical professionals and the general public repeatedly urged the Hong Kong government to close the Hong Kong-mainland border to stop further influx [4]. However, some questioned the effectiveness of such measure as there was already sign of local transmission in Hong Kong. They believed that border restriction is not useful in the presence of established local transmissions as the final disease burden might be primarily driven by local transmission instead of importing of foreign cases. While the COVID-19 situation is well controlled in 2021, there has been an urge to re-open the Hong Kong-mainland border to allow resumption of business activities. Yet, the Hong Kong and mainland China governments are hesitant on this.

Hong Kong is a Special Administrative Region of the People’s Republic of China and border control exists between the two regions. Owing to the tight geographical and socio-economic ties, more than forty-million individuals travelled from mainland China to Hong Kong annually [5]. There were also more than 200,000 Hong Kong citizens travelling daily to mainland China before the COVID-19 pandemics [6]. Implementing border restrictions between Hong Kong and mainland China has significant implication in both social and economic aspects. As such, there has been great debate on this policy since early 2020.

The objective of this study is to assess the impact of border restriction on cumulative caseload and hospital occupancy with a metapopulation Susceptible-Exposed-Infectious-Recovered (SEIR) model with inspected migration. Projection of COVID-19 epidemiology in Hong Kong and mainland China will be performed as an illustration.

## Methods

In this study, a metapopulation SEIR model with inspected migration was applied to investigate the epidemiological characteristics of COVID-19 in Hong Kong, Guangdong and the rest of China (excluding Hubei) in the presence or absence of border restriction. Guangdong was separately analyzed from the rest of China because Guangdong province had significantly higher confirmed cases per population (11.7 per million) than the rest of China (excluding Hubei) (9.5 per million) as of 20th February 2020, also acknowledging that the travel policy was also different from other provinces. Hubei province, with the highest case density in China (1048.4 per million), was excluded from analysis as all Hubei-Hong Kong travel was banned after the Wuhan lockdown on 23rd January 2020. Real world data from 23rd January 2020 (First reported case of COVID-19 in Hong Kong) to 8th February 2020 was used. This study involves a development of statistical model using historical data and does not involve active intervention to subjects involved.

### Metapopulation SEIR model with migration

SEIR type models are commonly adopted to simulate epidemiology of infectious disease of a single region over time [7–10]. It is based on a system of ordinary differential equations (ODE) that governs the number of 4 types of individuals: susceptible (S), exposed but latent (E), infectious (I), and recovered (or death) (R). Conventional single-patch SEIR models are not suitable for studying the impact of border restriction of an emerging infectious disease. A modified metapopulation SEIR model with inspected migration was used in this study. In addition to simulating population migration, parameters such as efficiency of custom inspection in blocking infected travelers were also being incorporated. Details of the model were described in Table 1.

**Table 1:** Model Parameters

Parameter	Value	Rationale/ Assumption
Latent period	5.2 days	As reprorted in Li et al. [11]
Infective period	5.0 days	Time from symptom onset to establishing diagnosis, getting isolated and rendering effectively non-infectious in Hong Kong.
Initial maximal *R*_0_	2.2	As reported in Li et al. [11, 12]
Temperature at which *R*_0_ reduce to 0	25.0 degree Celsius	Novel coronavirus transmissibility Hypothesized to reduce as temperature rises [13]. Threshold set with reference to temperature in Hong Kong in 2003 when SARS subsided near summer.
Efficiency of Immigration Department in blocking visitors in latent period (*σ*)	50%	Assumed household close contact of infected individuals are all quarantined and non-household close contact are not quarantined.
Efficiency of Immigration Department in blocking visitors in infectious period (*θ*)	99%	Assumed temperature monitoring and compulsory health declaration process at Immigration Department is 99% efficient.
In-patient mortality rate (lower bound)	1.36%	As reported by Guan et al. [9].
In-patient mortality rate (upper bound)	4.3%	As reported by Wang et al. [1].

### Real life epidemiological data

The population sizes of Hong Kong, Guangdong and the rest of China (excluding Hubei) at the time of analysis were 75,241,000, 113,460,000 and 1,222,750,000 [14] respectively. As of 7th February 2020, there were 26, 1034 and 5787 cases of laboratory confirmed COVID-19 patients in the three region respectively according to Hong Kong Department of Health and China Centre for Disease Control (CDC) data [15].

### Model parameters

The mean incubation and infectious period were taken as 5.2 and 5.0 days respectively [16]. The basic reproduction number *R*_0_ was set to linearly reduce from initial value at 18.0 °C to 0 at 25.0 °C. The temperature threshold was set by referencing Hong Kong temperature in the summer of 2003 when Severe Acute Respiratory Syndrome (SARS), which was also caused by coronavirus, subsided. Temperature in the projected period was modelled based on 2019 data released by the Hong Kong Observatory [17]. To explore the effect of border crossing restriction, we conducted simulations with 200,000 and 0 individuals travelling from mainland China to Hong Kong per day. We assumed 70% were from Guangdong and 30% were from the rest of China (excluding Hubei), based on the previous data from Hong Kong Immigration Department [13]. Efficiency of Immigration Department in blocking visitors in latent period (1 − *σ*) was taken as 50% by assuming household close contact of infected individuals were all quarantined and non-household close contact were not quarantined. Efficiency of Immigration Department in blocking visitors in infectious period (1 − *θ*) was taken as 99% by assuming that body temperature monitoring and compulsory health declaration at the Immigration Department were 99% efficient. The listed model parameter is summarized in Table 1. Simulation with multiple initial *R*_0_ values was performed, starting from 2.2, down to effective reproduction number *R*_*E*_ 1.6 at 0.1 intervals.

### Isolation facility occupancy

The Hong Kong public health system had a maximum of 952 isolation beds in 490 isolation single rooms according to the data from Hospital Authority press conference on 1st March 2020. It was assumed that all isolation facilities were used exclusively for COVID-19 purposes [18].

## Results

### Effect of complete border closure on case number and mortality

**Fig. 1:**
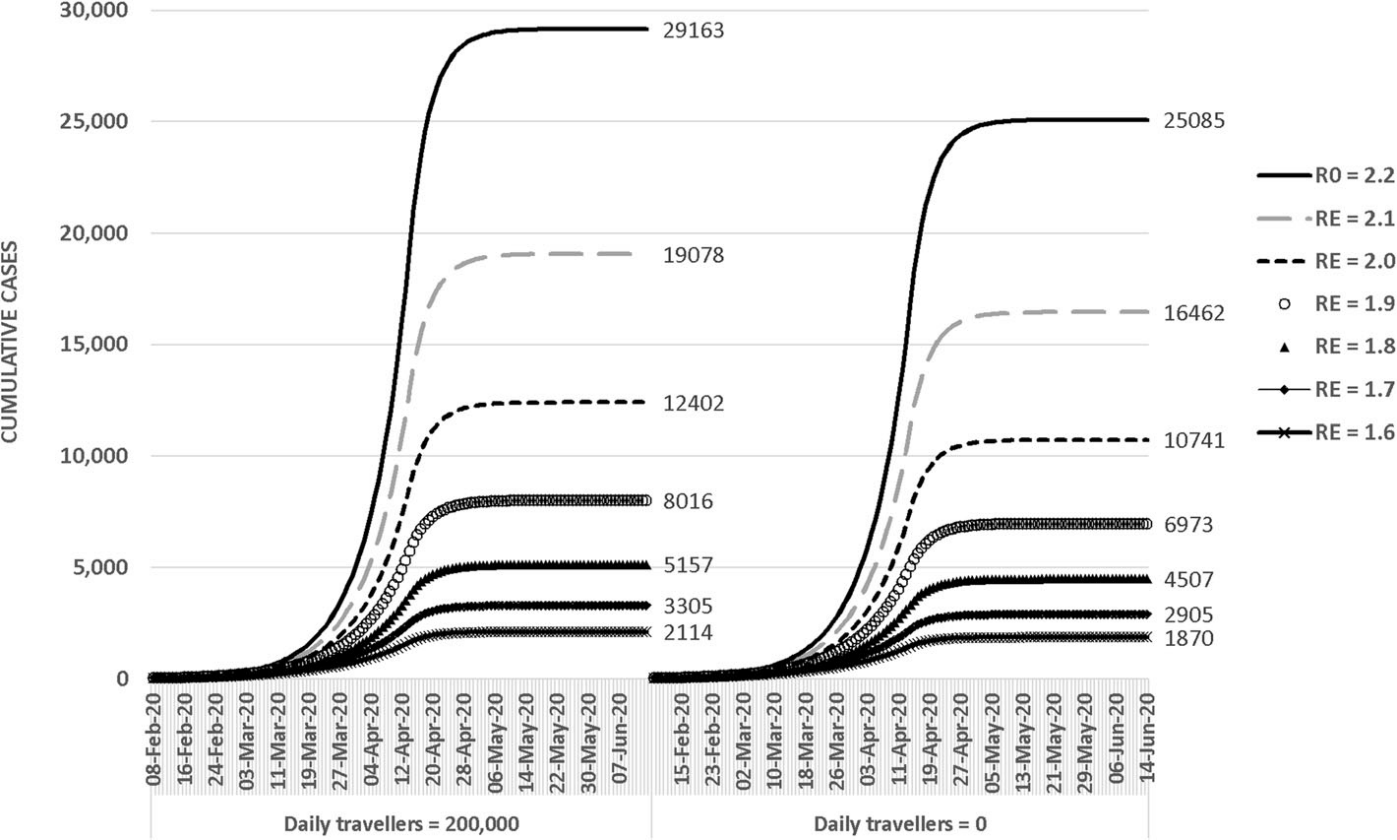
Effect of complete border closure on projected cumulative COVID-19 case number under differet *R*_0_/*R*_E_

We applied the metapopulation SEIR model with inspected migration to project the case number in the presence or absence of complete border closure. At *R*_0_ of 2.2, reduction in number of daily travelers from 200,000 to 0 starting 8th February 2020 would decrease the cumulative COVID-19 cases in Hong Kong by 13.99% from 29,163 to 25,084. At an in-patient mortality of 1.4% [13, 19]], the number of deaths can be reduced from 408 to 351 (57 lives saved). At *R*_0_ of 1.6–2.1, complete border closure was projected to cause a 11.54–13.71% reduction in cumulative cases and mortality (Fig. 1 and Table 2). The results suggested that even in the presence of established local transmission, travel restriction remained an effective measure to reduce the cumulative cases in the recipient region. COVID-19 associated mortality can also be decreased with this measure.

**Table 2:** Effect of complete border closure on the projected cumulative COVID-19 case & mortality at *R*_0_ = 2.2 and different *R*_0_ down to 1.6

	Without border closure (Daily traveller 200,000)	Complete border closure (Daily traveller 0)			
		Case reduction	Death averted		Percent reduction
			(@4.30%)	(@1.36%)	
*R*_0_ 2.2	29,163	4079	175	56	13.99%
*R*_*E*_ 2.1	19,078	2616	112	35	13.71%
*R*_*E*_ 2.0	12,402	1661	71	23	13.39%
*R*_*E*_ 1.9	8061	1088	45	14	13.50%
*R*_*E*_ 1.8	5157	650	28	9	12.60%
*R*_*E*_ 1.7	3305	400	17	5	12.10%
*R*_*E*_1.6	2114	244	11	4	11.54%

### Effect of public health measures on projected isolation facility demand

**Fig. 2:**
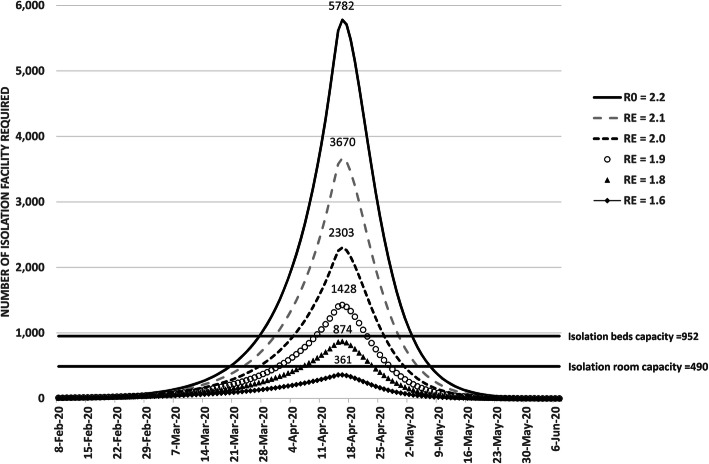
Effect of changes in reproductive number *R*_0_/*R*_E_ on isolation facility requirements over time

Local *R*_0_ of an infectious disease is partially dependent on effectiveness of public health measures implemented in a region. It can be in the form of contract tracing and quarantine system, or social distancing policies such as school cessation. For Hong Kong, at *R*_0_ of 2.2, the projected number of concurrent isolation facilities required to accommodate all infected individuals at the peak of the epidemic is 5,782 even with border restriction; this translated into the additional need of 5,292 new isolation rooms. In order to prevent the facilities in Hong Kong being overloaded, maintaining complete border closure and having effective public health measures to keep *R*_0_ below 1.6 are both required. These public health measures include universal masking, aggressive social distancing, suspension of school, work-from-home policy and temporary closure of recreational business and bars. Other permutations are shown in Table 3, and graphically represented in Fig. 2.

**Table 3:** Projected isolation facility deficit at *R*_0_ = 2 · 2 and different *R*_0_ down to 1·6 (Assuming complete border closure & 100% isolation / hospitalization rate)

	Maximum concurrent facility needed*	Additional isolation facilities required^			
		Single rooms		Isolation beds	
		Extra rooms needed	Date of reaching 100% occupancy	Extra beds needed	Date of reaching 100% occupancy
*R*_0_ 2.2	5782	5292	21-Mar-2020	4830	28-Mar-2020
*R*_*E*_ 2.1	3670	3180	24-Mar-2020	2718	31-Mar-2020
*R*_*E*_ 2.0	2303	1813	28-Mar-2020	1351	05-Apr-2020
*R*_*E*_ 1.9	1428	938	01-Apr-2020	476	10-Apr-2020
*R*_*E*_ 1.8	874	384	07-Apr-2020	N/A	N/A
*R*_*E*_ 1.7	528	38	14-Apr-2020	N/A	N/A
*R*_*E*_ 1.6	314	N/A	N/A	N/A	N/A

*^ Current capacity with 490 isolation rooms & 952 isolation beds*

## Discussion

In our study, a metapopulation SEIR model with inspected migration was applied to project the case number in the presence or absence of complete border closure. Complete border closure to travelers can reduce the cumulative COVID-19 case number in Hong Kong by 13.99% and mortality by 13.98%. The results suggested that even in the presence of established local transmission, travel restriction remained an effective measure to reduce the cumulative cases in the recipient region. In order to prevent overloading the isolation facilities in Hong Kong, apart from complete border closure, implementation of other effective public health measures to keep *R*_0_ below 1.6 would also be required.

Countries or cities with a high population density and aged population including Hong Kong is at risk of severe outbreak of emerging infectious diseases such as COVID-19 [20]. As the disease is spreading rapidly in multiple continents, many countries implemented border restrictions towards regions with severe outbreak in order to reduce local case number and mortality [20–22]. This is particularly important for developing countries with inadequate medical resources to tackle massive local outbreak. However, the WHO advised against utilizing travel restriction as an infection control measure [2]. Furthermore, it is particularly challenging to implement border restriction in certain regions due to political, social and economic reasons. To date, there is inadequate scientific data to support border restriction as a public health measure to limit the scale of local outbreak in the presence of an established local transmission. Using Hong Kong and mainland China as an example, we quantitatively illustrated border restriction is effective in reducing cumulative caseload, mortality and healthcare facility occupancy with a metapopulation SEIR model with inspected migration. It was projected that complete border closure would result in meaningful reduction of cumulative cases (4079 cases at *R*_0_ of 2.2), mortality (57 deaths at 1.4% in-patient mortality) and a delay in isolation facility overload in Hong Kong.

It is important to emphasize that in our projection, border closure alone is insufficient to prevent healthcare overload, as measured by isolation facilities occupancy. Effective and targeted public health intervention to slow local transmission and reduce local *R*_0_ is needed. It can be in the form of universal usage of surgical mask, contact tracing and quarantine system, or social distancing policies such as school cessation [23, 24]. The outbreak on Princess Diamond Cruise clearly illustrated the limitation in outbreak control by border restriction solely with no public health intervention [25, 26]. In early 2020, a number of passengers on Princess Diamond Cruise were found to have COVID-19. Despite there was no further import of COVID-19 cases onto the cruise after the immediate quarantine, there was still rapid rise in the number of COVID-19 cases on the cruise. It was believed that insufficient on-board personal protective equipment and inadequate social distancing were the causes of the unfortunate event. Of the 3711 individuals on the cruise, 624 of 3011 tested passengers were diagnosed with COVID-19 (16.7%) [25]. Unfortunately, implementation of strict public health measures may not be feasible to combat COVID-19 in many regions. For instance, social distancing may not be feasible due to environmental, economic, cultural or religion reasons. There may be a shortage of trained personnel and facilities for performing contact tracing and quarantine [23].

In the past 1 year, multiple regions had exponential rise in COVID-19 cases which caused extreme stress to their local health care system [11, 27]. In Wuhan, which was the epicenter of the COVID-19 outbreak in China, severe shortage in isolation facilities has necessitated the urgent construction of multiple temporary hospitals [12]. COVID-19 related mortality in regions with severe outbreak tend to be higher due to relative shortage of medical resources outweigh demand [28, 29]. Advanced life support facilities such as intensive care unit, ventilators, extracorporeal membrane oxygenation (ECMO) machines and anti-viral medications are essential in severe COVID-19 cases but their availability is limited [28, 29]. In addition, COVID-19 also severely hinders other non-COVID-19 related medical services. In Hong Kong, although the total confirmed COVID-19 cases are less than the available isolation facilities at the moment, a significant proportion of other less urgent medical services include elective investigations and surgeries have been suspended to reserve resources for COVID-19 [30]. In less resourceful regions, the effect may even be more pronounced. Although morbidity and mortality caused by such service suspension are not included in the official COVID-19 statistics, the effects cannot be overlooked. Furthermore, uncontrolled local epidemic can cause outbreaks in other regions with close ties [31]. The damage brought by a severe local outbreak of COVID-19 is unbearable. Therefore, it is paramount for governments around the world to prevent or limit scale of local outbreak. As suggested by our projection, border restriction against regions with severe outbreak could reduce local caseload, mortality and isolation facilities occupancy. Furthermore, aggressive and efficient public health measures to reduce local *R*_0_ is necessary [32].

The study finding have important implication on policy making. While the COVID-19 pandemic has not ended and many countries made progress in COVID-19 vaccination, how to fine-tune the border restriction should be based on scientific decision. This can be done by using this metapopulation SEIR model with inspected migration to estimate the risks of loosening the border restrictions between different countries and areas. This could inform a risk-based re-opening of the border for resumption of social and economic activities. Incorporating this metapopulation SEIR model with inspected migration into public health policy making would allow timely and scientific decision on border restriction measure, which is an area of ongoing debate nowadays, when there is an urge for opening the borders to facilitate the resumption of economic activities.

### Strength of the model

The spread of infectious disease is closely related to the migration of population between regions [13, 18]. Conventional single-patch SEIR models are not suitable for such analysis. A metapopulation SEIR model with inspected migration was specifically applieded for this purpose. In addition to COVID-19, the developed model can be used to perform projection for other emerging infectious diseases in the future [33]. Furthermore, parameters such as effectiveness of custom inspection were included to improve accuracy of projection. The presented model is also suitable for further analysis of other emerging infectious diseases.

### Limitation

Firstly, interaction was assumed to be well-mixed within patch. The spatial effect in disease transmission within each patch is not directly addressed in the model, which can have a non-trivial effect on the dynamic of infectious disease [34]. Secondly, the proposed model is deterministic in nature which ignores the randomness in migration and in the interactions among people; a stochastic model would be more realistic especially early in the disease [35, 36]. Thirdly, key parameters such as rate of spread are still unclear so we assumed a parametric form of the rate of spread with reference to 2003-SARS [37]. In general, parameter calibration can be performed by some criteria [38], for example, minimizing residuals sum of square between the historical and fitted infected cases. Meanwhile, missing information, such as travel history across regions, leads to crucial statistical uncertainty. A stochastic metapopulation migration model to explore the corresponding statistical properties with data would be a fruitful direction in the future [36]. While the above shortcomings may be the expected tradeoff between computation time and model simplicity [39], it will show the core message that border restriction reduces cumulative case, mortality and delay healthcare system exhaustion. Lastly, economic impact is beyond the scope of this study [40]. While full border closure can have a negative impact on the economy, one cannot ignore the negative economic impact from an otherwise preventable major outbreak.

## Conclusion

This study showed that early implementation of travel restriction is effective in reducing cumulative cases and mortality in Hong Kong. However, additional public health measures such as mandatory masking and social distancing to reduce local R_0_ to below 1.6 are required to prevent COVID-19 from overwhelming hospital isolation facilities.

## Data Availability

All data generated or analyzed during this study are included in this published article.

## Abbreviations

COVID-19: Coronavirus Disease 2019
SARS-CoV-2: Severe Acute Respiratory Syndrome CoronaVirus 2
MERS-CoV: Middle East Respiratory Syndrome CoronaVirus
ARDS: Acute Respiratory Distress Syndrome
WHO: World Health Organization
SEIR: Susceptible-Exposed-Infectious-Recovered
CDC: China Centre for Disease Control
ECMO: Extracorporeal membrane oxygenation
