# Correction to: Modelling the impact of travel restrictions on COVID-19 cases in Hong Kong in early 2020

**DOI:** 10.1186/s12889-021-12157-x

**Published:** 2021-11-17

**Authors:** Wang-Chun Kwok, Chun-Ka Wong, Ting-Fung Ma, Ka-Wai Ho, Louis Wai-Tong Fan, King-Pui Florence Chan, Samuel Shung-Kay Chan, Terence Chi-Chun Tam, Pak-Leung Ho

**Affiliations:** 1 Department of Medicine, Queen Mary Hospital, Hong Kong, SAR China; 2 Department of Statistics, University of Wisconsin, Madison, USA; 3 Department of Astronomy, University of Wisconsin, Madison, USA; 4 Department of Mathematics, Indiana University, Bloomington, USA; 5 Department of Microbiology and Carol Yu Centre for Infection, University of Hong Kong, Hong Kong, SAR China

**Correction to: BMC Public Health (2021) 21:1878.**

**https://doi.org/10.1186/s12889-021-11889-0**

It was highlighted that the in the original article the name of Chun-Ka Wong was incorrectly shown as Ka-Chun Wong. The original article has been updated [1].
